# The psychiatry virtual-on-call experience: Can it improve confidence of foundation and GP trainees with out-of-hours work in psychiatry?

**DOI:** 10.1192/bjo.2021.130

**Published:** 2021-06-18

**Authors:** Charlotte Harrison, Helen Blamey, Alistair Roddick, Kate Saunders, Tina Malhotra

**Affiliations:** 1 OUCAGS (Oxford University Clinical Academic Graduate School), University of Oxford, Oxford Health NHS Foundation Trust; 2 OUCAGS (Oxford University Clinical Academic Graduate School), University of Oxford, Department of Psychiatry, University of Oxford, Warneford Hospital; 3 Oxford Health NHS Foundation Trust

## Abstract

**Aims:**

Out-of-hours (‘on-call’) work can be perceived by junior doctors to be a daunting experience, associated with feeling unprepared and less supported. Simulated on-call programmes have been used to great effect in medicine and surgery to improve junior doctors’ skills in task prioritisation, interpersonal communication and confidence on-call. However, few psychiatry-specific programmes exist.

We aimed to: i) Develop a psychiatry specific virtual-on-call programme, ii) Investigate if the virtual-on-call programme improved confidence amongst junior trainees in key areas of psychiatry practice.

**Method:**

The Psychiatry Virtual-On-Call programme commenced in December 2020. It involves attending an introductory on-call lecture, followed later in the rotation by a 2-hour simulated on-call shift. All trainees are expected to attend during their attachment and the simulated shifts are ongoing. During the shift, trainees are ‘bleeped’ with different psychiatry specific tasks. They work through the tasks, using local intranet policies and telephone advice from the on-call psychiatry registrar. Due to COVID-19 the sessions were delivered virtually. Participants completed a questionnaire evaluating confidence in ten domains, rated on a Likert scale from 0–10. Questionnaires were completed at four time-points during the programme; pre- and post-introductory lecture and pre- and post-simulated shift. Scores were compared using Mann-Whitney U tests. Significance was defined as P < 0.05 with Bonferroni correction applied for multiple testing.

**Result:**

Twenty-nine trainees attended the introductory lecture, 25 and 21 trainees completed the pre- and post-lecture questionnaire respectively. A non-significant improvement in confidence was reported in three domains: seclusions reviews, prescribing, detention under the mental health act.

At the time of writing, ten trainees had attended the on-call shift. All participants completed a pre- and post-session questionnaire. The on-call shift was a useful learning experience (median score 9), and significantly increased perceived preparedness for on-call work from 3/10 to 7/10 (p < 0.001). Confidence was significantly improved in seven domains, most markedly in seclusion reviews, prescribing and mental health act tasks.

**Conclusion:**

The psychiatry virtual-on-call programme fills a niche in the training curriculum and is perceived by trainees to be a useful learning experience. The introductory lecture improved confidence in several domains, but not as effectively as the on-call shift. The on-call shift was well received by participants and significantly improved confidence in 7/10 domains. In summary, the virtual-on-call experience improves preparedness for out-of-hours psychiatry work. Follow-up of participants at the end of their psychiatry rotation will ascertain if they felt the programme to be useful during out-of-hours work.

